# Tracheal Compression on Chest X-ray Leading to the Diagnosis of Right Aortic Arch in 22q11.2 Deletion Syndrome

**DOI:** 10.7759/cureus.93404

**Published:** 2025-09-28

**Authors:** Masahito Kachitori, Rina Tsuchiya, Ryuta Orimoto, Dai Kuranobu, Kentaro Miyai

**Affiliations:** 1 Department of Pediatrics, Tokyo-kita Medical Center, Tokyo, JPN

**Keywords:** 22q11.2 deletion syndrome, circumflex aorta, hypocalcemia, right aortic arch, tracheal compression

## Abstract

22q11.2 deletion syndrome (22q11.2DS) is a relatively common disorder in pediatrics, frequently presenting with immune dysfunction, cardiac malformations, and hypoparathyroidism, all stemming from the development of the fourth branchial arch. While intracardiac malformations, such as tetralogy of Fallot, are highly prevalent, aortic abnormalities are also common. We report the case of a male diagnosed with 22q11.2DS at 12 years of age. His medical history included neonatal hypocalcemia, developmental delays, and later-onset tetany due to hypoparathyroidism. Despite a normal echocardiogram, a chest X-ray revealed deviation of the right tracheal wall, prompting MRI evaluation that identified a right aortic arch. This report highlights the diverse phenotypic spectrum of 22q11.2DS, demonstrating that significant vascular anomalies originating from the fourth branchial arch, such as right aortic arch, can occur even in the absence of classic intracardiac malformations. It underscores the importance of a comprehensive diagnostic approach, including advanced imaging modalities such as MRI, especially when subtle signs of external tracheal compression are evident on chest X-ray.

## Introduction

22q11.2 deletion syndrome (22q11.2DS) is a relatively common pediatric disorder, often characterized by immune dysfunction, cardiac malformations, hypocalcemia due to hypoparathyroidism, and mild facial dysmorphisms [1]. These manifestations are primarily attributed to abnormal development of organs derived from the fourth branchial arch [1,2]. Intracardiac malformations, particularly tetralogy of Fallot, are highly prevalent, affecting more than half of individuals with 22q11.2DS [3]. Given the critical role of the fourth branchial arch in conotruncal development, aortic abnormalities are also frequently observed in patients with 22q11.2DS [4]. We describe the case of a 12-year-old male diagnosed with 22q11.2DS. His medical history included neonatal hypocalcemia, global developmental delay, and later-onset tetany due to hypoparathyroidism. Notably, chest radiography revealed deviation of the right tracheal wall. Although echocardiographic findings were unremarkable, subsequent MRI confirmed the presence of a right aortic arch.

## Case presentation

Our patient was a male born at 39 weeks of gestation via normal vaginal delivery, with a birth weight of 2,090 g (-3.02 SD), height of 45 cm (-1.93 SD), and head circumference of 32.5 cm (-0.53 SD). On the seventh day after birth, tremors were noted in his extremities, prompting admission to another hospital. Brain CT, electroencephalogram, and blood tests revealed no significant abnormalities except for mild hypocalcemia (7.8 mg/dL). The tremors gradually subsided without specific treatment, and he was discharged shortly thereafter. At 10 months of age, he came to our hospital due to mild delays in motor ability and intellectual development. He was later diagnosed with autism spectrum disorder and was followed up until the age of six years.

**Figure 1:**
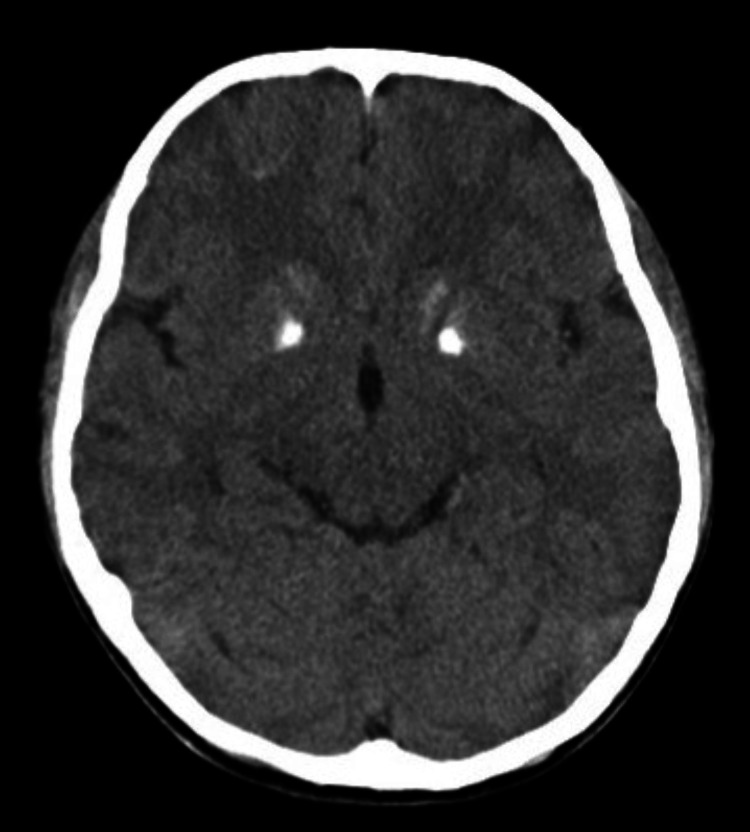
Brain CT at admission Bilateral high-density areas in the basal ganglia are observed CT: computed tomography

**Figure 2:**
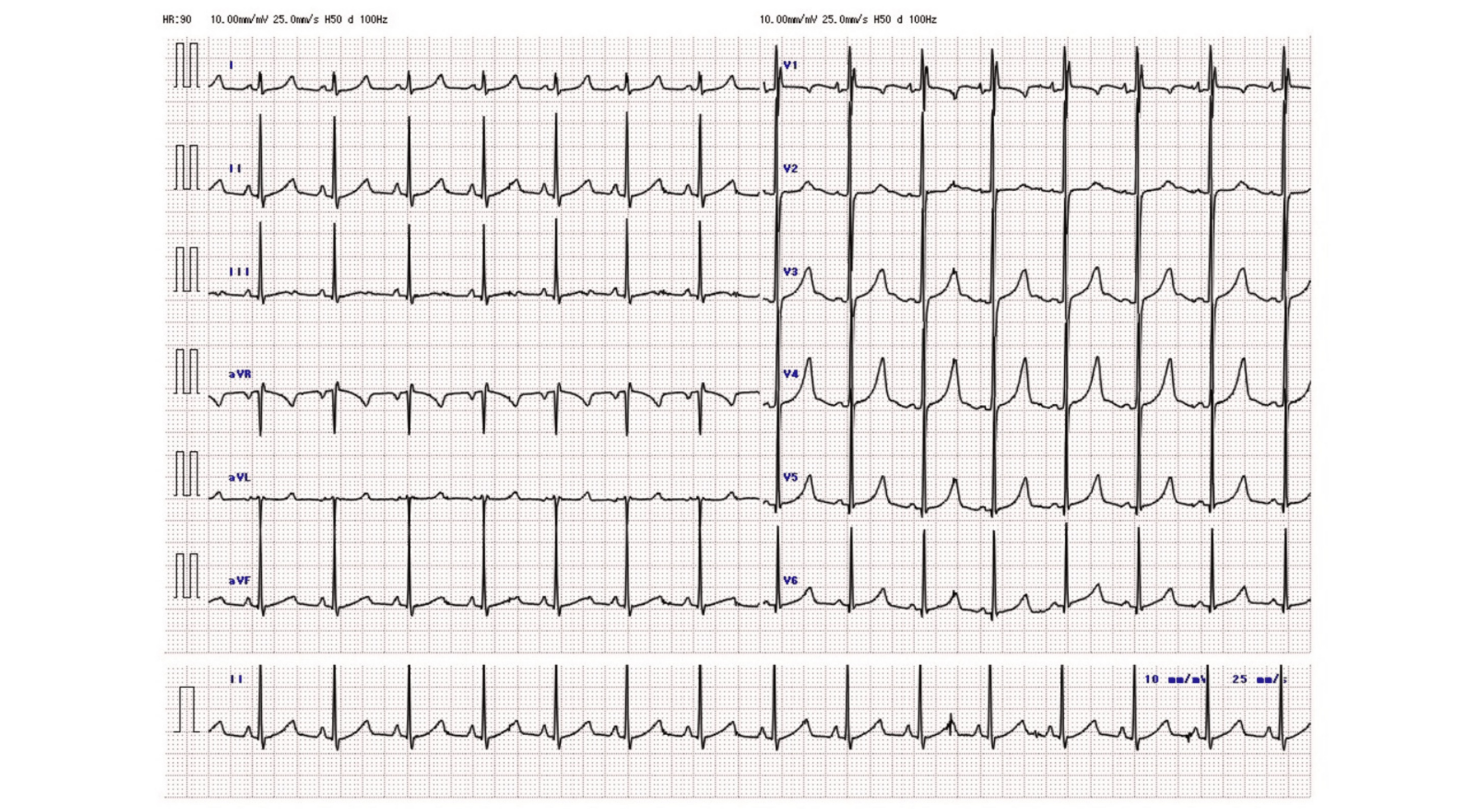
Electrocardiogram at admission QT interval and corrected QT interval are 0.464 seconds and 0.524 seconds, respectively

At 12 years of age, he returned to our hospital with intermittent left-sided rigidity. Brain CT revealed bilateral basal ganglia calcification (Figure 1). ECG was normal except for a prolonged QTc interval (0.524 seconds) (Figure 2). Blood tests showed hypocalcemia (6.8 mg/dL) and low intact parathyroid hormone (PTH) (14 pg/mL), as well as low urinary calcium to creatinine ratio (0.0005 mg/mg) and normal levels of 25-OH Vitamin D and serum magnesium, leading to the diagnosis of tetany secondary to hypoparathyroidism (Table 1). His penile and pubic hair were both Tanner stage 4, and his testes volume was 20 mL for each, suggestive of puberty. The symptoms of tetany resolved following intravenous calcium gluconate administration. Oral calcium lactate and alfacalcidol were also initiated, and his serum calcium levels gradually normalized without recurrence of tetany. Calcium gluconate was discontinued seven days later, and he was discharged on the 10th day of hospitalization.

**Table 1:** Summary of laboratory data PTH: parathyroid hormone; 25(OH)D: 25-hydroxyvitamin D; 1,25(OH)2D: 1,25-dihydroxyvitamin D

Parameter	Patient value	Reference range
Calcium, mg/dL	6.5	8.8 - 10.1
Phosphate, mg/dL	8.3	2.7 - 4.6
Magnesium, mg/dL	1.8	1.8 - 2.4
intact-PTH, pg/mL	14	10 - 65
25(OH)D, ng/mL	23.2	>20
1,25(OH)2D, pg/mL	111	20 - 60

**Figure 3:**
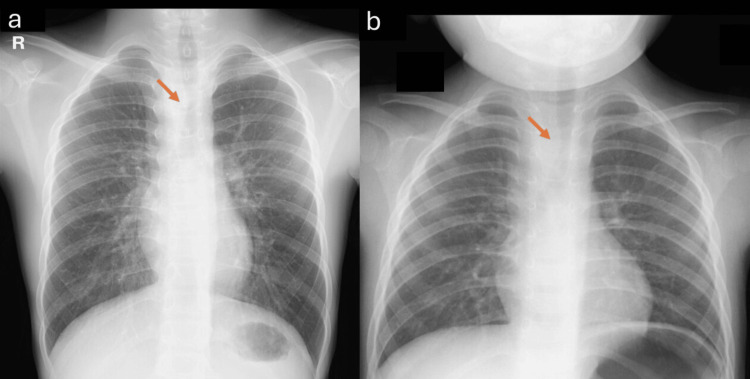
Chest X-rays at the age of 12 years (a) and three years (b) Both X-rays show right side tracheal wall compression (arrow). The thoracic descending aorta seems to be on the left side of the spine

**Figure 4:**
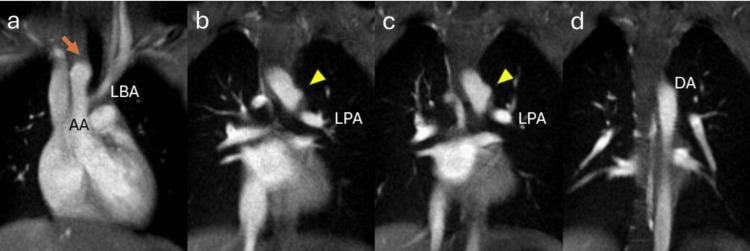
Coronal sections of chest MRI a. The ascending aorta compresses the right tracheal wall (arrow). b. The bulge at the anterior-left side of the proximal descending aorta is found (arrowhead). c. The bulge seems to connect to the left pulmonary artery. d. The thoracic descending aorta is on the left side of the spine MRI: magnetic resonance imaging; AA: ascending aorta; LBA: left brachiocephalic artery; LPA: left pulmonary artery; DA: descending aorta

**Figure 5:**
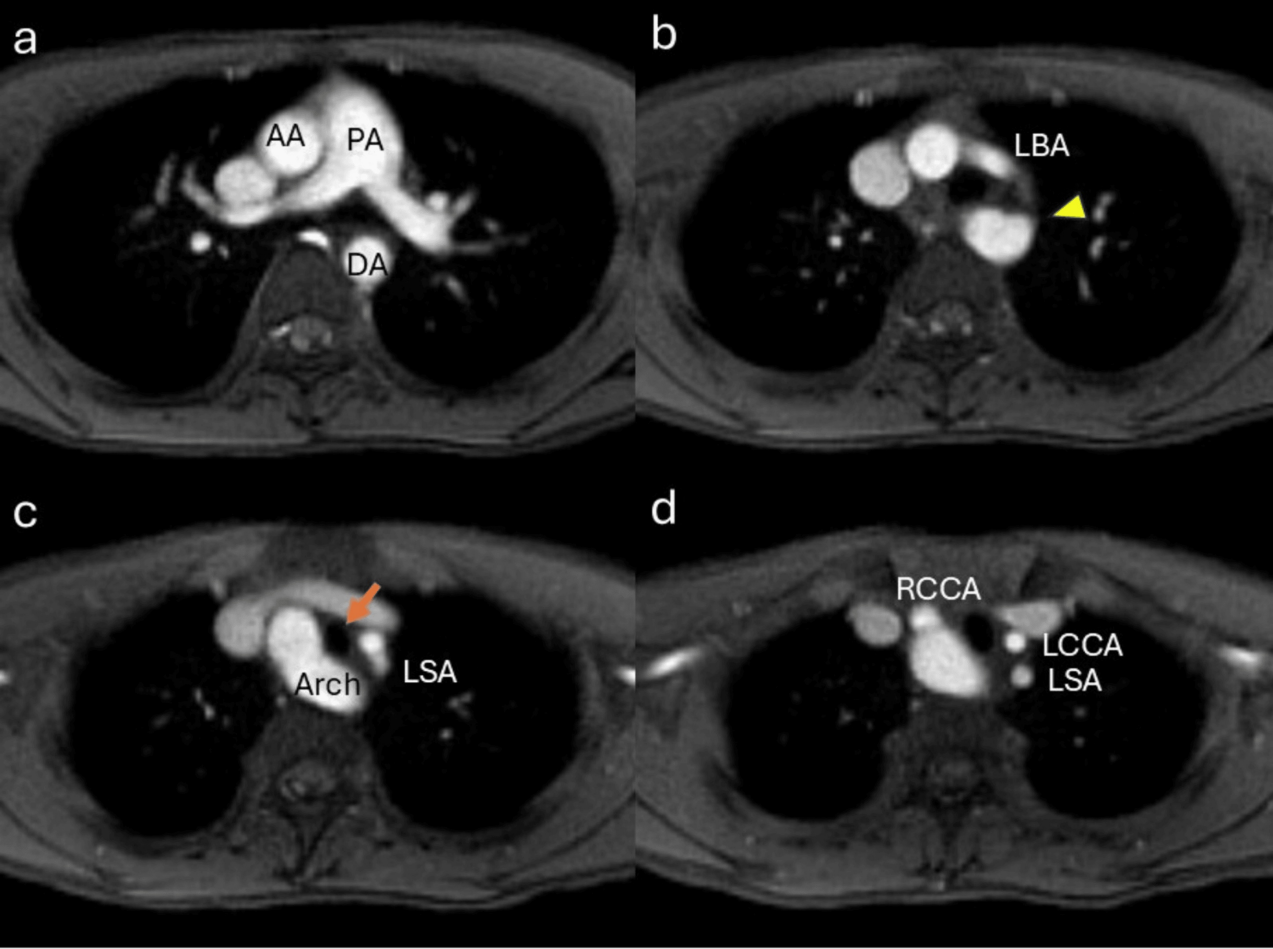
Axial sections of chest MRI a. The aortic arch is on the right side of the pulmonary artery, and the descending aorta is on the left side of the spine. b. The ascending aorta debranches the left brachiocephalic artery (LBA). The bulge is found on the anterior-left side of the descending aorta (arrowhead). c. The aortic arch traverses from the right side to the left side, posterior to the trachea (arrow). The trachea is shorter in diameter from side to side. d. The ascending aorta debranches the right common carotid artery, and the LBA bifurcates the left common carotid artery and the left subclavian artery MRI: magnetic resonance imaging; AA: ascending aorta; PA: pulmonary artery; DA: descending aorta; LBA: left brachiocephalic artery; Arch: aortic arch; LSA: left subclavian artery; RCCA: right common carotid artery; LCCA: left common carotid artery

A chest X-ray taken at admission revealed deviation of the mid-right tracheal wall (Figure 3a). This deviation had also been noted on a chest X-ray taken at the age of three during hospitalization for acute bronchitis (Figure 3b). Though he exhibited no symptoms related to tracheal or esophageal compression and had a normal echocardiogram, an MRI was performed to investigate the cause of the tracheal wall deviation. MRI revealed a right aortic arch in close proximity to the right side of the trachea (Figures 4a, 5a), causing deviation without significant compression. No severe tracheal stenosis was observed. The MRI also showed that the right aortic arch branched the left brachiocephalic artery first, followed by the right common carotid artery and the right subclavian artery (Figures 5b, 5c, 5d). The right aortic arch coursed from the right to the left behind the trachea and esophagus, superior to the carina, in a configuration known as right circumflex aorta, connecting to the descending thoracic aorta on the left side of the spine (Figures 4d, 5b, 5c). Additionally, a bulge of the left side of the proximal descending aorta was noted (Figures 4b, 4c, 5b).

**Figure 6:**
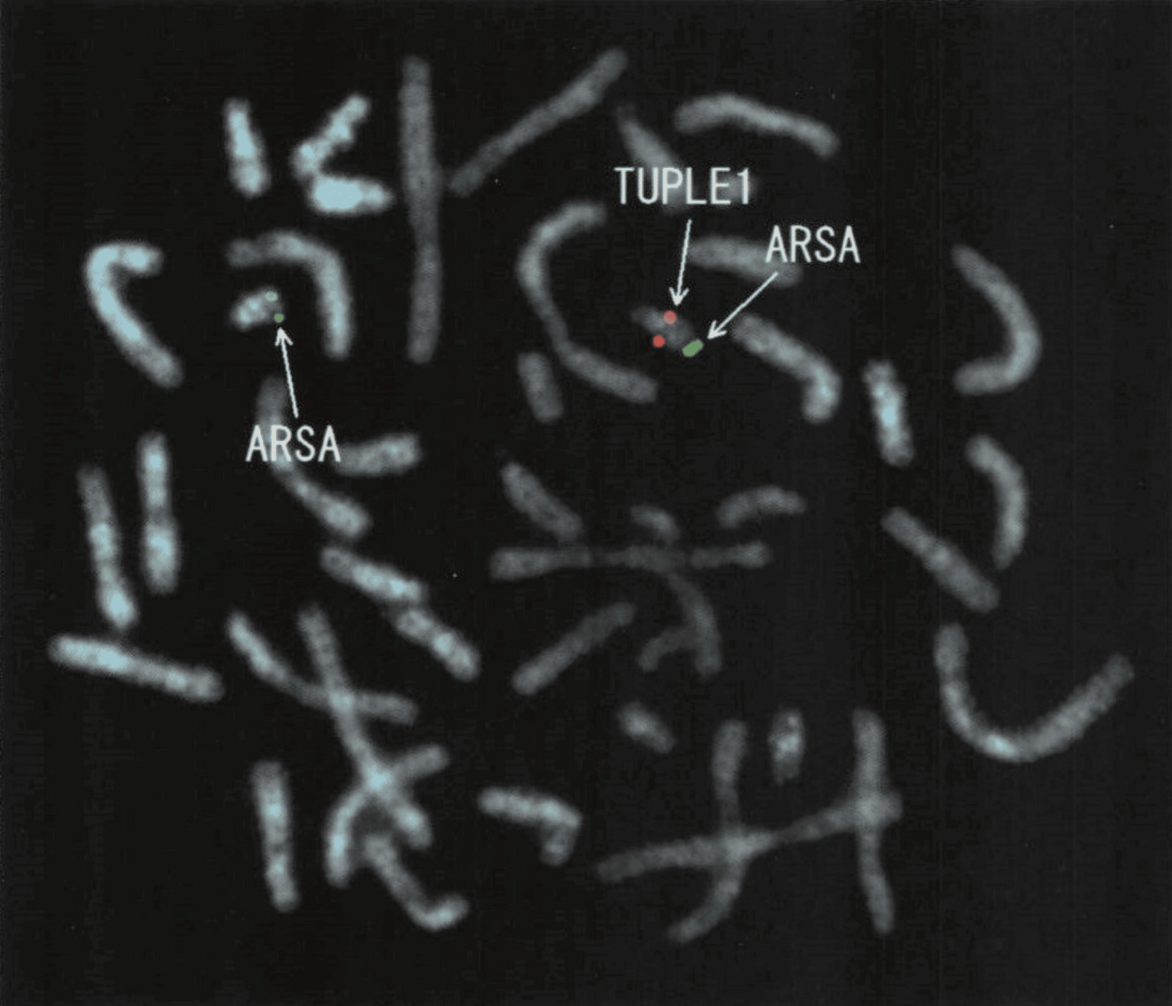
Image of FISH analysis TUPLE1 and ARSA probes are labelled as red and green, respectively. Both genes exist in the 22q region, and the absence of the red signal indicates deletion of the TUPLE1 locus at 22q11.2 FISH: fluorescent in situ hybridization

Given the patient’s history of tetany, seizure-like episode with hypocalcemia in the neonatal period, right aortic arch with tracheal wall deviation, alongside mild facial dysmorphism (small jaw, small mouth, small ears, and small eyes), 22q11.2DS was suspected. After obtaining informed consent from his parents, FISH analysis was performed, confirming the 22q11.2 deletion (Figure 6). Although congenital heart diseases are frequently associated with 22q11.2DS, his echocardiogram showed no significant abnormalities.

## Discussion

In our patient, the deviation of the right tracheal wall observed on chest radiography served as the initial clue leading to the diagnosis of right aortic arch. Although typical radiographic findings of right aortic arch include rightward displacement of the aortic arch, right-sided descending aorta, or leftward deviation of the trachea, none of these features were evident in this patient [5,6]. The only abnormality noted was the deviation of the right tracheal wall. In cases without intracardiac anomalies, a right aortic arch is often challenging to visualize using echocardiography, particularly in asymptomatic individuals. Therefore, in syndromes such as 22q11.2DS, which are frequently associated with cardiovascular anomalies, even subtle radiographic findings warrant further evaluation using advanced imaging modalities such as MRI.

A right aortic arch may form a vascular ring through specific branching patterns or persistence of the ductus arteriosus, potentially leading to tracheoesophageal compression and associated clinical symptoms [7]. In this case, the right aortic arch exhibited a rare configuration known as right circumflex aorta, in which the arch courses from the right to the left side and connects to the descending thoracic aorta. If a left ductus arteriosus was present during fetal development, it may have formed a vascular ring via the ligamentum arteriosum. Although the patient currently exhibits no symptoms of airway or esophageal compression, the anatomical configuration poses a risk for future complications such as respiratory distress or dysphagia [8]. Accordingly, careful longitudinal monitoring is warranted, and surgical intervention may be considered when the symptoms arise.

In this case, the left brachiocephalic artery branches off from the right aortic arch, followed by the right common carotid artery and the right subclavian artery, classified as right aortic arch with mirror-image type [9]. In addition, the connection of the arch to the descending aorta on the left side classifies it as right circumflex aorta [9,10]. While there have been several reports of right circumflex aorta in children, they are often associated with coarctation or ventricular septal defects, and cases without hemodynamic abnormalities, such as this case, are rare [11,12].

The bulge observed in the descending aorta corresponds to the attachment site of the ligamentum arteriosum (Figures 4b, 4c). Furthermore, this bulge may represent the regressed segment of the left aortic arch after branching the left common carotid artery and the left subclavian artery (Figure 5b). Anatomically, the left brachiocephalic artery and the descending aorta are in close proximity (Figure 5b, 5c). Regardless of its exact etiology, the attachment site of the ligamentum arteriosus is a potential predisposition for traumatic aortic rupture [13]. Thus, it might be important to monitor this bulge for potential aneurysm formation.

Congenital heart disease is highly prevalent in individuals with 22q11.2DS, occurring in 60% to 80% of cases [3]. Common cardiac anomalies include tetralogy of Fallot, interrupted aortic arch, and persistent truncus arteriosus [14]. Although less common, a right aortic arch has also been reported in individuals with 22q11.2DS [3,15]. These cardiac anomalies are closely linked to defects of conotruncal development. Since 22q11.2DS disrupts the normal development of the fourth branchial arch, it frequently results in outflow tract abnormalities [14]. Therefore, it is essential to evaluate both intracardiac and conotruncal abnormalities thoroughly in patients diagnosed with 22q11.2DS.

Hypocalcemia most likely manifests during the neonatal and adolescent periods, primarily due to the increased calcium demand during periods of highly active bone growth [16,17]. In healthy individuals, PTH secretion regulates blood calcium levels. However, individuals with insufficient PTH secretion or PTH action are prone to hypocalcemia in such periods. In 22q11.2DS, transient hypocalcemia may occur during the neonatal period but often remains asymptomatic due to compensatory dietary intake. Nevertheless, hypocalcemia may become symptomatic during adolescence, even in the absence of neonatal cardiac or immune abnormalities, as observed in this case [18]. Therefore, when hypocalcemia due to hypoparathyroidism is diagnosed around puberty, it is essential to screen underlying conditions such as 22q11.2DS.

Phenotypic variability in 22q11.2DS cannot be fully explained by hemizygosity of the deleted region alone [1]. The 22q11.2 locus contains multiple low-copy repeat sequences, which predispose the region to non-allelic homologous recombination, resulting in chromosomal deletions. These deletions vary in size, ranging from the typical ~3 Mb deletion to smaller microdeletions [1]. Notably, phenotypic differences have been reported even among family members carrying the same deletion, suggesting that factors beyond gene dosage contribute to the clinical heterogeneity [19]. This underscores the importance of maintaining a broad differential diagnosis and anticipating a wide range of clinical manifestations once 22q11.2DS is identified.

## Conclusions

This case report highlights a critical aspect of 22q11.2DS: its diverse phenotypic expression. We emphasize that patients with this syndrome can present with vascular anomalies originating from the fourth branchial arch, such as a right aortic arch, even in the absence of the more commonly recognized intracardiac malformations. This finding underscores the importance of a comprehensive diagnostic approach that extends beyond echocardiography, incorporating advanced imaging techniques such as MRI, particularly when subtle signs of tracheal or esophageal compression are observed.
